# Implementation of an ISO 15189 accredited next generation sequencing service for cell-free total nucleic acid (cfTNA) analysis to facilitate driver mutation reporting in blood: the experience of a clinical diagnostic laboratory

**DOI:** 10.1136/jcp-2024-209514

**Published:** 2024-06-24

**Authors:** Reiltin Werner, Ruth Crosbie, Mairead Dorney, Amy Connolly, Dearbhaile Collins, Collette K Hand, Louise Burke

**Affiliations:** 1 Pathology Department, Cork University Hospital, Cork, Ireland; 2 Department of Pathology, School of Medicine, University College Cork College of Medicine and Health, Cork, Ireland; 3 Department of Medical Oncology, Cork University Hospital, Cork, Ireland

**Keywords:** Pathology, Molecular, DIAGNOSIS, DNA

## Abstract

**Aims:**

Next generation sequencing (NGS) on tumour tissue is integral to the delivery of personalised medicine and targeted therapy. NGS on liquid biopsy, a much less invasive technology, is an emerging clinical tool that has rapidly expanded clinical utility. Gene mutations in cell-free total nucleic acids (cfTNA) circulating in the blood are representative of whole tumour biology and can reveal different mutations from different tumour sites, thus addressing tumour heterogeneity challenges.

**Methods:**

The novel Ion Torrent Genexus NGS system with automated sample preparation, onboard library preparation, templating, sequencing, data analysis and Oncomine Reporter software was used. cfTNA extracted from plasma was verified with the targeted pan-cancer (~50 genes) Oncomine Precision Assay (OPA). Assessment criteria included analytical sensitivity, specificity, limits of detection (LOD), accuracy, repeatability, reproducibility and the establishment of performance metrics.

**Results:**

An ISO 15189 accredited, minimally invasive cfTNA NGS diagnostic service has been implemented. High sensitivity (>83%) and specificity between plasma and tissue were observed. A sequencing LOD of 1.2% was achieved when the depth of coverage was >22 000×. A reduction (>68%) in turnaround time (TAT) of liquid biopsy results was achieved: 5 days TAT for in-house analysis from sample receipt to a final report issued to oncologists as compared with >15 days from reference laboratories.

**Conclusion:**

Tumour-derived somatic variants can now be reliably assessed from plasma to provide minimally invasive tumour profiling. Successful implementation of this accredited service resulted in:

Appropriate molecular profiling of patients where tumour tissue is unavailable or inaccessible.

Rapid TAT of plasma NGS results.

**WHAT IS ALREADY KNOWN ON THIS TOPIC**

- Implementing an ISO 15189 accredited next-generation sequencing (NGS) service for liquid biopsy in a clinical diagnostic histopathology laboratory is a novel innovation that is not routinely available in clinical diagnostic pathology laboratories. Recent significant developments in NGS technologies, platforms and automated workflows have enabled this clinical diagnostic laboratory in a designated tertiary National Cancer Control Programme cancer centre to establish an accredited cell-free total nucleic acids/liquid biopsy NGS service.

**WHAT THIS STUDY ADDS**

- This study provides an NGS implementation roadmap for clinical diagnostic pathology departments that have increased demands for advanced diagnostics of DNA and fusions via NGS on blood samples.

**HOW THIS STUDY MIGHT AFFECT RESEARCH, PRACTICE OR POLICY**

- Patients undergoing targeted therapy for lung cancer can have their treatment monitored via blood sampling, allowing early and highly sensitive detection of mutations related to acquired resistance. Incorporating liquid biopsy NGS into the clinical diagnostic workflow allows for enhanced diagnostics, improvements in targeted treatments and cancer trials for an expanded cohort of patients. Thus ensuring appropriate use of healthcare resources, which will ultimately lead to improved outcomes for patients with cancer.

## Introduction

Molecular pathology techniques, and more specifically, next generation sequencing (NGS) technologies, are integral to the delivery of personalised medicine to patients with cancer.^1–3^ The rate of treatment development, in addition to the rapid increase in demand for emerging novel types of biomarkers, has led to the selection of multigene NGS assays as the preferred methodology for targeted analysis of tumour samples.^4 5^

In advanced non-small cell lung cancer (NSCLC), it has been suggested that up to 69% of patients harbour oncogenic driver alterations that are actionable or are linked to an approved treatment, with response rates of 56%–74% in those that received targeted treatment.^6 7^ Though approximately 25% of patients do not have tissue that is accessible or available for traditional tissue biopsy molecular testing, there is still a requirement to establish the molecular status of these patients with cancer.^8–11^ Liquid biopsy via peripheral blood sampling can facilitate this genomic profiling.^12 13^ In addition, comprehensive molecular analysis in patients with NSCLC is imperative when they present with progressive disease to guide subsequent treatment and management decisions.^14–17^ However, this analysis can be challenging using only traditional tissue biopsy in this cohort of patients due to the effects of their prior therapy.^18 19^ Not all cancer foci are easily accessible and the ability to biopsy may be affected by the risk of bleeding or pneumothorax.^20^ The complexities of ‘tumour heterogeneity’ are also now a concept of increasing significance in treatment decision processes.^21 22^ This phenomenon encompasses the fact that the biopsied tumour might not be fully representative of the entire tumour or metastatic disease elsewhere.^23 24^ Therefore, the choice of the best treatment option for patients with driver mutations requires a deep and repeated investigation of the evolving molecular environment using methodologies that can capture and analyse these complexities and heterogeneity,^25 26^ such as liquid biopsy.

A comparison of traditional tissue and liquid biopsy was presented by Lone *et al*, and the main advantages are that it is a minimally invasive procedure for the patient is highly sensitive and has a faster turnaround time (TAT). Therefore, it offers a convenient, fast and precise approach to identify targetable oncogene mutations and resistance mechanisms in addition to, and sometimes instead of, the traditional methods.^27–30^ Although initially primarily used for the detection of *EGFR* T790M NM_005228.5(EGFR):c.2369C>T mutations linked to acquired resistance to therapy in NSCLC, the application has been expanded to the early identification of other emerging resistance mutations, such as *EGFR* C797S NM_005228.5(EGFR):c.2390G>C.^31–34^ This highlights the importance of tracking mutations throughout the disease course, as it is essential to timely clinical decision-making and treatment planning.^25 35^

While there are advantages to NGS with liquid biopsy, there have been issues with the determination of the status of fusions when using cell-free DNA (cfDNA)-based methods.^36–38^ The challenges of identifying gene fusions in liquid biopsy have been reported with greater sensitivity when using RNA-based NGS.^39^ Some circulating cell-free RNA (cfRNA) assays have reached over 77% sensitivity, though the preanalytical conditions play a significant role in ensuring this rate of detection.^40^ The assay under evaluation in this study is a cell-free total nucleic acid (cfTNA)-based assay encompassing DNA and fusions with a targeted Oncomine panel on the Ion Torrent Genexus. While Low *et al*’s study outlines a comparable sequencing workflow, they conducted their off-instrument cfTNA extraction separately using the Applied Biosystems MagMax kit.^41^ Thus, to our knowledge, this is the first report to assess the performance of the fully automated Genexus cfTNA NGS workflow, from sample preparation, extraction and quantitation on the Genexus Purification Instrument (GPI), library preparation, template preparation, sequencing and data analysis on the Genexus Integrated Sequencer, to reporting with Oncomine Reporter in a clinical diagnostic laboratory.

### Objectives

The Ion Torrent Genexus has previously been accredited to ISO 15189 with formalin-fixed paraffin-embedded (FFPE) tissue.^42^ The Genexus may also be used for cfTNA isolated from plasma without requiring a high level of user expertise in bioinformatics and reporting.^43^ It facilitates a minimal nucleic acid input volume of approximately 5 ng, with run sizes of 1–5 cfTNA samples, using a cost per assay instead of a cost per run model.

A prerequisite when optimising new techniques in clinical diagnostic molecular laboratories is compliance with national and international guidelines^17 44–46^ and European Society for Medical Oncology recommendations.^28^ In this setting, optimal clinical validation and accreditation are paramount^47 48^ and were the principal components of this project.

The following key objectives were identified:

1. Optimisation and verification of methodology and automated platforms for plasma sequencing.
2. Establish 5-day TATs for plasma/cfTNA NGS results.
3. Attain ISO 15189 accreditation (INAB ISO15189:2012) for plasma/cfTNA.

## Materials and methods

This study assessed the performance of a targeted panel, the Oncomine Precision Assay (OPA) (Thermo Fisher Scientific) for hotspot genes and fusions for clinical application in plasma testing, in a controlled and phased manner.

### Preanalytical phase: sample selection and collection

NGS validation was performed across two Genexus sequencing platforms with total samples (n=60), including real-world clinical NSCLC samples (n=29), commercial reference material (Thermo Fisher Scientific TFS and Horizon Diagnostics) (n=20) and External Quality Assessment (EQA) proficiency testing samples (Genomics Quality Assessment (GENQA), European Molecular Genetics Quality Network (EMQN) and Quality in Pathology (QUIP)) (n=11). Real-world plasma samples were used with either matched clinical (FFPE) tumour samples previously characterised via an NGS assay or matched plasma samples tested with PCR methods onsite. Between 10 and 16 mL of blood were collected in K2/K3 EDTA tubes. Roche cfDNA tubes (Roche Diagnostics) were also verified (n=11) for use; however, the results were excluded from comparative analysis as plasma samples from these tubes are not suitable for long-term storage (>30 days) at −80 °C. Samples were centrifuged at 2000*g* at 4°C for 10 min×2 to separate plasma within 0.5–4 hours of sample collection for EDTA and within 5 days for cfDNA tubes. The plasma samples were stored at −80°C until sample preparation on the Genexus Purification Instrument (GPI).

Commercial reference material supplemented the real-world samples for a robust evaluation of assay performance. Reference materials are homogenous, well-established controls for the calibration and validation of diagnostic instruments.^49^ Proficiency testing with EQA samples was performed and evaluated by accredited external quality assurance schemes (GENQA, EMQN and QUIP).

### Sample preparation

Off-instrument preprocessing was performed as per manufacturer instructions (Ion Torrent Genexus cfTNA purification kit (part No. A45535)) with proteinase K added just prior to nucleic acid extraction; this took approximately 30 min.

Extraction and purification steps were automated on the GPI over 14 runs with minimal hands-on time (15 min). The GPI has a built-in Qubit for fluorimetric quantitation. Quantified TNA, with a minimum of 5 ng nucleic acid input, was prepared on an archive plate and transferred directly onto the Genexus plate and sequencer.

### Library preparation, sequencing and data analysis

The OPA was the primary assay validated and evaluated for use in this study; however, the Oncomine Dx Express test (ODxET) was verified as an orthogonal assay. These targeted pan-cancer panels encompass variants across ~50 key genes, including mutations, copy number variants (CNVs), fusion variants and hotspot mutations (substitutions, insertions and deletions; see table 1).

**Table 1:** Oncomine assays target genes

DNA hotspots			Copy number variants	Fusions	
AKT1	ESR1	MAP2K2	ALK*	ALK	NTRK2
AKT2	FGFR1	MET	AR	BRAF	NTRK3
AKT3	FGFR2	MTOR*	CD274*	ESR1	NUTM1
ALK	FGFR3	NRAS	CDKN2A*	FGFR1	RET
AR	FGFR4	NTRK1	EGFR	FGFR2	ROS1
ARAF	FLT3	NTRK2	ERBB2	FGFR3	RSPO2
BRAF	GNA11*	NTRK3	ERBB3	MET	RSPO3
CDK4	GNAQ*	PDGFRA	FGFR1	NRG1	AR
CDKN2A*	GNAS	PIK3CA	FGFR2	NTRK1	EGFR
CHEK2	HRAS	PTEN	FGFR3		
CTNNB1	IDH1	RAF1	KRAS		
EGFR	IDH2	RET	MET		
ERBB2	KEAP1†	ROS1	PIK3CA		
ERBB3	KIT	SMO*	PTEN*		
ERBB4	KRAS	STK11†			
	MAP2K1	TP53			

All samples were processed on the Genexus Purification Instrument and Genexus sequencers with automated library preparation, sequencing and bioinformatic analysis (Genexus software V.6.6, Thermo Fisher Scientific).

*Oncomine precision assay only

†Oncomine Dx Express test only

### Reporting

Oncomine Reporter (OR) (software V.5.8, Thermo Fisher Scientific) is a genomic analysis tool developed specifically for downstream analysis of NGS data and the generation of complete reports, including the stratification of variants and recommendations for therapy and clinical trials. The database is monitored, with software updated monthly following a review of label guidelines (National Comprehensive Cancer Network, European Medicines Agency andFood and Drug Administration)^46 50 51^ and changes to available clinical trials. Variant call files from Genexus were uploaded to the OR software, and reports were generated for authorisation by the consultant pathologist.

### TAT measurements

TAT was established as the request date for a complete molecular report, defined as a consultant pathologist-authorised, integrated report of NGS results visible in the electronic medical record to the treating clinician. TAT was calculated in business days.

### Accreditation to ISO 15189

An application was made to the Irish National Accreditation Board (INAB) to add this NGS service for plasma cfTNA samples to the annual accreditation assessment schedule. It was audited to ISO 15189 standards by the external body following validation.

### Analytical validation

Laboratory-specific performance metrics were established for OPA, including limits of detection (LOD), measurement uncertainty, minimal depth of coverage, minimum read counts, mapped reads, uniformity and variant allelic frequency.

The Oncomine panel was assessed to determine positive percentage agreement (PPA) and positive predictive value (PPV) for each variant type. Assessment parameters included determining analytical sensitivity, specificity, LOD, accuracy, reproducibility, inter-lot, inter-operator and inter-run variability.

### Establishment of quality performance metrics

In line with guidelines for validation of NGS panels,^48^ the number of assays performed (n=60) enabled the accurate establishment of test performance characteristics. Like previous studies,^47^ the quality and depth of coverage metrics were measured across all clinical validation specimen data sets to establish acceptable run-level quality control parameters. These performance metrics included the percentage of reads mapped to the reference sequence for the DNA and RNA libraries. A minimum threshold for each performance metric was established for ongoing quality control.

## Results

**Figure 1:**
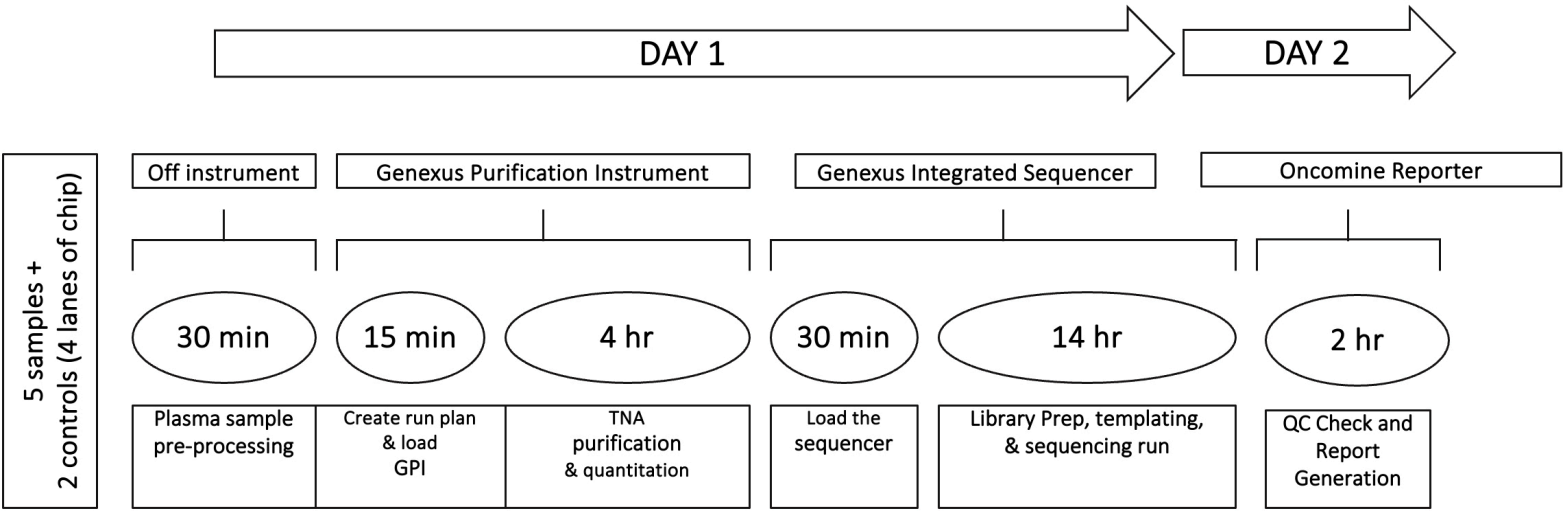
Schematic of the NGS cfTNA processing workflow established in-house with a 2-day TAT and minimal hands-on time. cfTNA, cell-free total nucleic acid; NGS, next-generation sequencing; TAT, turnaround time.

This is a multifaceted project carried out over 6 months, from initial concept to ISO 15189 accreditation. NGS testing was carried out as per the schematic in figure 1, depicting the sample processing to report workflow. Results and metrics are based on the OPA panel evaluation, as the ODxET was used for verification only.

### Nucleic acid extraction and quantification

**Figure 2:**
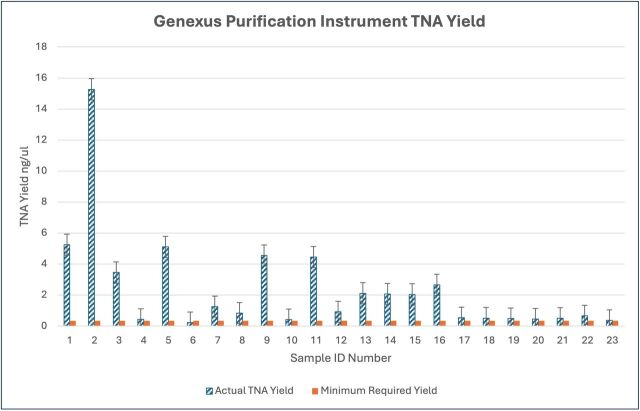
TNA yield of real-world patient samples quantified on Genexus Purification Instrument plasma cfTNA extraction versus minimum required yield according to the manufacturer’s specifications (0.33 ng/uL) for Oncomine assay next-generation sequencing. cfTNA, cell-free total nucleic acid.

The verification of the GPI cfTNA protocol was carried out by extracting, purifying and quantifying the TNA from 29 real-world plasma samples and commercial/EQA material (n=31), followed by sequencing on the Genexus. TNA quantification results from the GPI are available on 23 patient samples run on the GPI prior to NGS; six real-world samples not quantified on board the GPI were also sequenced successfully. TNA yield is dependent on several factors, including the amount of DNA circulating in the liquid biopsy and sample quality or appropriate preanalytical steps.^52^ The minimum manufacturer-based threshold for progressing to sequencing is 0.33 ng/uL (5 ng TNA); however, one sample (sample 6 in figure 2) with a low TNA input of 3.3 ng (0.22 ng/uL) was sequenced successfully, detecting a confirmed *EGFR* Exon 20 insertion with an allele frequency of 8.8%.

### Sequencing performance

Run success was based on passing performance metrics established over the course of the optimisation. Data collated from the Genexus software enabled the generation of performance metrics, similar to quality parameters established previously for tissue NGS on the Genexus.^42^ These internal quality control checks determine which findings are released when curating reports (table 2). Importantly, the mapped reads for the DNA library according to manufacturer specifications should be between 8 and 12 million reads per sample. Another significant metric is the variant allelic fraction detected, which corresponds to the fraction of sequencing reads harbouring a mutation.^53^

**Table 2:** Plasma cfTNA next-generation sequencing performance metrics established at optimisation.

Oncomine performance metrics cfTNA	
Metric	Target
Final reads	10–12 million/Lane
Raw read accuracy	97%–99%
% Loading	88%–92%
Enrichment	99.90%
Library	99.90%
Mapped reads/DNA library	8–12 million
% Reads on target	>90%
Mean read coverage	22 000–40 000
Uniformity	97–99%
Mean molecular coverage	1000–3000
Variant allelic fraction	>1.2%/0.012
MAPD	<0.4 (0.14–0.25)
Mean read length DNA	99–100
Mapped reads/RNA library	> 150 000–400 000
Mean read length RNA	97–104
RNA detection	>2/7
Base call accuracy	97%–99%

cfTNA, cell-free total nucleic acid; MAPD, Median Absolute Pairwise Difference.

### OPA performance: variant detection accuracy

In line with the objective of establishing this testing for patients with NSCLC, the verification included real-world plasma samples from patients with lung cancer. The 11 currently reportable or Tier 1/actionable variants for NSCLC (*ALK, EGFR, BRAF, KRAS, NTRK1, NTRK2, NTRK3, ROS, RET, MET* and *ERBB2*) were verified and commercial control material was utilised to supplement the verification. The 50 gene panel included a range of variants beyond NSCLC variants; these were included in the verification to allow for the future expansion of the plasma NGS Oncomine assay for use in other cancer types.

The real-world samples included two blood tube types: EDTA (K2/K3) and Roche cfDNA tubes. The EDTA tubes had a higher level of concordance with the orthogonal methods. While they must be transported immediately to the laboratory (<4 hours), they are suitable for long-term storage once the plasma is prepared and stored at −80°C. The Roche cfDNA tubes hold stability for 1 week; however, the manufacturer recommends plasma storage at −80°C for <30 days. The discordance in the samples stored in cold storage for 12 months could be due to inappropriate storage and degradation. Therefore, the samples collected in the Roche cfDNA tubes were removed from the comparative analysis.

True positives, false positives and false negatives (FN) were determined for each sample in the targeted regions meeting the minimum quality requirements. The overall concordance to orthogonal methods with PPA and PPV in this validation is 83%. Three discordant real-world samples did not detect confirmed variants in the tissue biopsy. In sample S05, the tissue biopsy had two *EGFR* variants present; only the *EGFR* EX19DEL was detected in plasma, with *EGFR* T790M not detected. Similarly, a *KRAS* G12D was detected in tissue only (sample S14), and an *ALK* Fusion was detected in the FFPE sample only (sample S11).

To ascertain the presence of homopolymer regions or positional effects in all samples, the variant calls underwent visual inspection in the Integrative Genomic Viewer (IGV) software. This examination aimed to identify whether any calls were located within homopolymer regions or proximate to the end of a read. Upon scrutiny, it was found that neither positional effects nor homopolymer effects were evident.

### OPA analytical reproducibility, sensitivity and specificity

**Figure 3:**
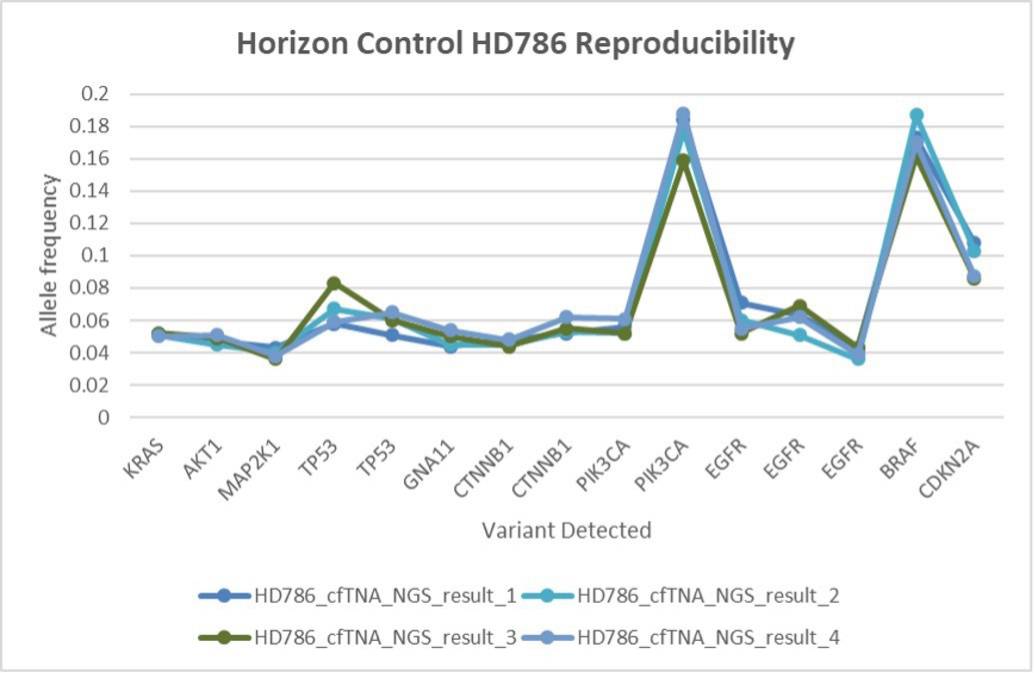
The reproducibility of cfTNA testing was established over four runs using the Structural Multiplex cfDNA Reference Standard HD786 (Horizon Diagnostic). The 15 variants present in HD786 exhibited allele fractions within the expected manufacturer reference ranges. HD786 includes a range of structural variants, that is, translocations, fusions, copy number variants and large insertion - deletions (INDELs). cfTNA, cell-free total nucleic acid; NGS, next-generation sequencing.

The analytical specificity of small DNA variants was assessed by correctly identifying samples that do not harbour any of the variants being profiled. Good reproducibility was demonstrated by performing sequencing on control material across four runs with different operators, reagents and chips on different days, with all results consistent and within range (figure 3).

**Figure 4:**
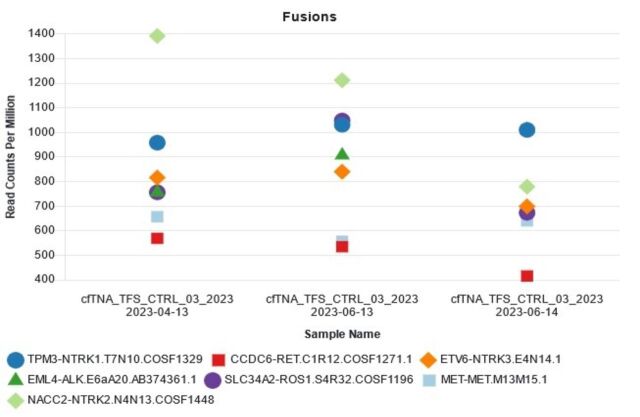
Fusion reproducibility assays: plasma cfTNA control (cfTNA_TFS_CTRL) (Thermo Fisher Scientific) compared over three runs on three different days by different operators, detecting all seven key non-small cell lung cancer fusions (*TPM3-NTRK1, CCDC6-RET, ETV6-NTRK3, EML4-ALK, SLC34A2-ROS1, MET-MET.M13M15* and *NACC2-NTRK2*) above minimum expected read counts (>400 read counts per million). cfTNA, cell-free total nucleic acid.

The availability of real-world samples limited fusion analysis; however, standardised reference controls were repeatedly tested for RNA fusions. All expected NSCLC key fusion variants were correctly identified in the artificial samples, conferring an assay sensitivity of 100%. In line with the expected results for specificity, only the seven fusions (*ALK, ROS, RET, MET, NTRK1, NTRK2* and *NTRK3*) were detected in these samples. Intra-run and inter-run reproducibility was assessed by testing three replicates of the plasma cfTNA control (Thermo Fisher Scientific) over three runs, with excellent concordance between and within runs (figure 4).

**Figure 5:**
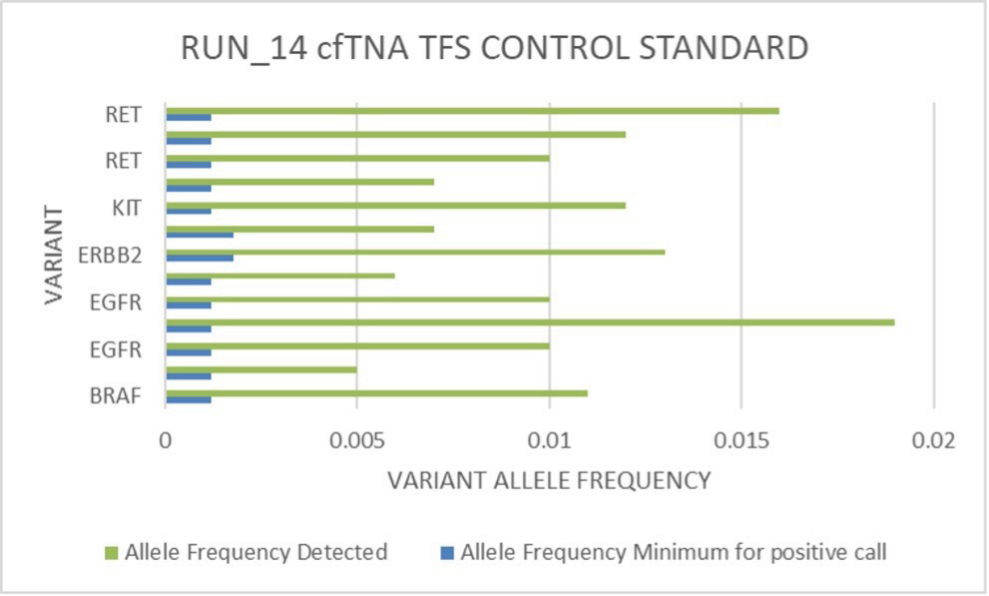
Plasma cfTNA control (cfTNA_TFS_CTRL) (Thermo Fisher Scientific), allelic fraction for run 14. Variants included in this commercial control (*RET, KIT, ERBB2, EGFR* and *BRAF*) allele frequency detected (green) with a minimum expected variant allelic fraction (blue) for a positive call according to the manufacturer’s specifications. cfTNA, cell-free total nucleic acid.

In addition to the real-world samples, commercial reference samples (n=20) were used over 14 runs (see figure 5); the TFS reference standard variants detected exceeded the manufacturer-established minimum threshold for a positive call.

**Figure 6:**
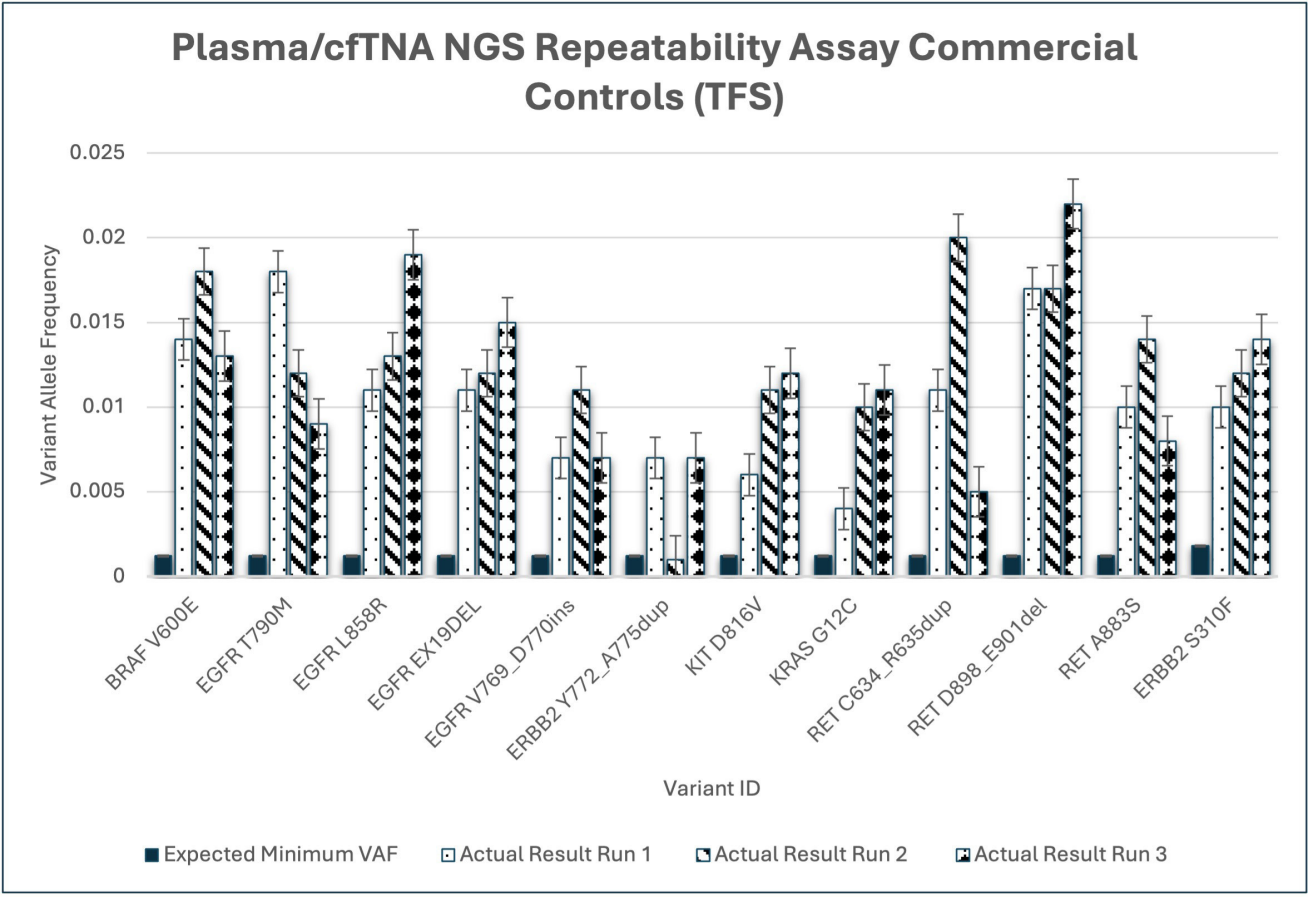
Repeatability assay with plasma cfTNA control (Thermo Fisher Scientific) commercial control; Oncomine NGS variant detected over 3× runs, including expected minimum variant allele frequency according to manufacturer’s specifications (minimum VAF of 0.0012). cfTNA, cell-free total nucleic acid; NGS, next-generation sequencing; VAF, variant allelic fraction.

Repeatability was consistently demonstrated using the plasma TNA control (Thermo Fisher Scientific; see figure 6); the variant allelic fraction detected in runs 1, 2 and 3 versus the expected manufacturer-based minimum allelic fraction (AF) threshold of 0.0012 AF indicates that each variant is present above this minimum required baseline.

**Figure 7:**
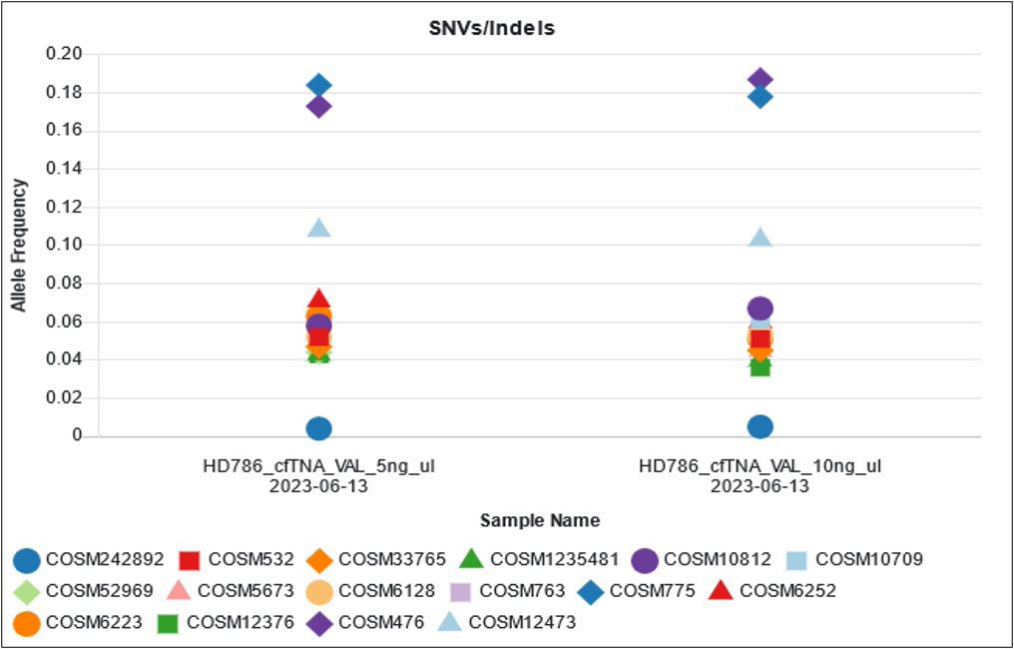
HD786 Horizon Multiplex Reference Standard dilution studies for Single Nucleotide Variants (SNVs) / Insertion - deletions (Indels). Commercial control (HD786) diluted to concentrations of 0.5× in the 10 ng/uL sample and 0.25× in the 5 ng/uL sample.

Good assay specificity and sensitivity were further demonstrated by dilution studies of control samples at concentrations of 0.5 and 0.25× (figure 7). Samples were analysed at varying dilutions, which facilitated the determination of LOD. While variant allelic fractions as low as 0.005 were detected, a 1.2%, or 0.012 LOD, was established when a depth of coverage of 22 000× was reached.

#### NGS Results Turnaround Time

A 6-month post-implementation audit of NGS cfTNA plasma TAT in-house was 5 days, compared with the target TAT of the outsourced reference laboratory of >15 working days, plus additional days for authorisation in-house by the consultant pathologist. Implementation of this system resulted in a 68% reduction in the TAT of liquid biopsy NGS results. The time and resources required for obtaining and processing a blood sample are also considerably less than those for a tissue biopsy.

#### Accreditation

The INAB audit thoroughly examined the NGS service, considering each of the (ISO 15189) standards. INAB accreditation was successfully attained.

## Discussion

The objective of establishing an ISO 15189-accredited NGS testing service for plasma/cfTNA from sample to report was accomplished within 6 months. Concurrently, efforts were made to reduce the TATs of NGS plasma results. The implementation of a fully integrated workflow not only minimised hands-on time, thus freeing up medical scientists, but also streamlined every step of the process through automation outlined in figure 1. The Genexus rapid sequencing technology provided results in a single day, an improvement over other systems.^42^ Integrating this liquid biopsy service on-site within the clinical diagnostic pathology department proved pivotal in enhancing TAT. While the full automation of Genexus facilitated this fast TAT, it was the transition from previous referral laboratory outsourcing that accounted for a remarkable 68% reduction. Efforts focused on optimising the automated NGS workflow for cfTNA/plasma further contributed to this enhancement. This feasibility study demonstrated a concordance rate of 83%, with variant detection rates achieved by orthogonal methods and matched FFPE tissue samples. However, it is worth noting that three discordant cases (samples 5, 11 and 14) failed to detect confirmed variants in the tissue biopsy. This discrepancy is likely due to the presence of low-abundance tumour genetic material circulating in the blood, with allelic frequencies below 1%, as also described by De Luca *et al* in their study.^54^ Where there is limited sensitivity of liquid biopsy (70%–80%) and no variant is detected, obtaining a tissue biopsy result where possible is recommended to avoid reporting FNs.^12^ A potential improvement to this workflow would be the possibility of ascertaining the tumour fraction, which is the proportion of total cfDNA in a sample that is tumour-derived (ctDNA), as a good marker of a genuine tumour signal. Thereby enhancing confidence in confirming a true negative plasma result and providing valuable guidance for timely treatment selection.^55^ Despite the discordant cases, the sensitivity and specificity of this assay remain comparable to those reported in other studies.^56 57^ An in-house post-implementation trend analysis of real-world clinical cfTNA samples (n=25) was found to have detection rates of between 60% and 65%, depending on the NSCLC stage and timing of the liquid biopsy. This suggests that the NGS workflow implemented on the Genexus platforms is effective in delivering accurate and timely results for plasma/cfTNA testing, with the potential to improve patient care and clinical outcomes significantly. However, there is still room for improved sensitivity, which is likely to be addressed by further research into advancing testing technologies and novel techniques in vivo. For instance, the future possibility of administering priming agents that protect ctDNA from destruction in the bloodstream is an interesting field of study that may augment levels of available ctDNA.^58^

The study highlighted the importance of standardised preanalytical protocols at the outset. Variations in assay results in the optimisation phase were linked to plasma sample storage time. The stability of blood samples fluctuated depending on the type of tube and prolonged storage at −80°C. The following key points should be noted:

- Sample collection: the differences between K2/K3 EDTA tubes, Roche cfDNA tubes and other blood tubes regarding stability and processing requirements. Understand the importance of timely processing for EDTA tubes (<4 hours) and the advantage of Roche cfDNA tubes for sample storage up to a week. Samples from EDTA tubes should ideally be processed within 1 hour to prevent contamination with cfDNA from leucocyte lysis.^59^ In contrast, the manufacturer guidelines state that for cfDNA collection tubes (Roche Diagnostics), samples can be stored at room temperature for up to 7 days. However, with cfDNA preservative tubes, contamination with leucocyte DNA may begin earlier, necessitating processing within 3 days.^60^ Even in samples processed immediately, tumour-specific somatic mutations typically represent <1% of the total cfDNA for the region of interest. Any increase in contamination with ‘diluting’ cfDNA from in vitro lysis could yield FN results.^61^ Therefore, it is recommended to perform a two-step centrifugation of EDTA samples before freezing and storage to reduce contamination from leucocyte DNA.
- Ensure immediate preprocessing is part of the defined workflow to maintain sample integrity.

One limitation of this study was the relatively small number of real-world clinical samples used (n=29). Future projects to build upon this research should include more fusions such as *ALK*, *ROS* and *MET*. While the study was supplemented with commercial control material, there remains a scarcity of real-world clinical samples with known fusions due to their low prevalence in NSCLC, which ranges from 0.9% to 5%.^62 63^ Similarly, CNV analysis requires further verification with real-world samples before implementing CNV testing and reporting in cfTNA. By expanding the sample pool, future studies can enhance the reliability and applicability of NGS plasma testing for various genetic alterations, thereby improving its clinical utility and impact on patient care.

## Conclusions

While FFPE tumour biopsy is the standard of care in precision medicine to guide treatment, a structured assessment of both tissue and blood samples is required to obtain a comprehensive molecular understanding of oncogenic mutations identified in cancer and the temporal evolution of these mutations. Identifying and tracking variants using minimally invasive blood draws helps overcome challenges associated with tumour heterogeneity and inadequate or low-quality tissue samples.

The fully automated cfTNA assay on the Genexus evaluated in this paper is cost-effective and time-efficient, making it a suitable aid for prognostication and determining the most appropriate individualised treatments. Incorporating this comprehensive testing with relative ease throughout the patient treatment journey allows for enhanced diagnostics, improvements in oncological possibilities and cancer trials for patients. Ensuring the appropriate use of healthcare resources will consequently lead to better outcomes for cancer patients .

## Data Availability

All data relevant to the study are included in the article or uploaded as supplementary information.
